# Supplementary material for: Short-term persistence of foliar insecticides and fungicides in pumpkin plants and their pollinators
Source: PLoS One. 2025 Apr 2;20(4):e0311634. doi: 10.1371/journal.pone.0311634 (PMC11964230; doi:10.1371/journal.pone.0311634)
Supplement: S1 Table — To preserve the anonymity of growers, farms are listed by identifiers A – E. The approximate location of each grower’s farm, rounded to the nearest 1/10th decimal degree for anonymity, is as follows: A – 40.1N, -82.8W; B – 40.0, -82.4; C – 40.1, -82.7; D – 40.0, -82.6; E – 39.9, -82.8. Spray events during the study period are numbered sequentially within farm. For example, at farm B spray event 1, the grower applied both carbaryl and permethrin on the same day. Pesticide residue names are given as their commercial formulations and % active ingredient(s). The rate of application is given in fluid ounces per hectare, either as a range or a single concentration, as reported by the growers. (PDF) [file pone.0311634.s001.pdf]

S1 Table. Summary of pesticides applied during the study period that we tested for in pumpkin and bee tissues.

		**Insecticides**			**Fungicides**		
**Grower**	**Spray Event**	**Sevin** (43% carbaryl)	**Perm-up** (36.8% permethrin)	**Province** (22.8% lambda cyhalothrin)	**Procure** (42.14% Triflumizole)	**Quintec** (22.58% Quinoxyfen)	**Bravo** (40.4% Chlorothalonil)
A	29 Jul	158.1 – 237.2	-	-	-	-	79.1 – 118.6
B	29 Jul	158.1 – 237.2	15.8 – 31.6	-	-	-	-
	7 Aug	79.1	-	-	-	-	-
C	24 Jul	158.1 – 237.2	-	-	-	-	79.1 – 118.6
	9 Aug	-	-	-	-	9.9 – 14.8	-
D	26 Jul	-	-	-	-	9.9	-
	3 Aug	-	-	-	14.8	-	-
E	26 Jul	-	-	6.3 – 9.5	-	-	79.1 – 118.6
Farms		3	1	1	1	2	3
Spray Events		4	1	1	1	2	3
