# Supplementary material for: Short-term persistence of foliar insecticides and fungicides in pumpkin plants and their pollinators
Source: PLoS One. 2025 Apr 2;20(4):e0311634. doi: 10.1371/journal.pone.0311634 (PMC11964230; doi:10.1371/journal.pone.0311634)
Supplement: S2 Table — (PDF) [file pone.0311634.s002.pdf]

S2 Table. Daily weather records during the study period from 23 July – 16 August, 2019.

Date	Spray event	Sampling event	max temperature C	mean temperature C	rainfall (mm)
23-Jul-19		X	25.6	20.74	1.78
24-Jul-19	X		25.6	20.23	0.00
25-Jul-19		X	27.2	21.53	0.00
26-Jul-19	X		28.9	23.10	0.00
27-Jul-19		X	30.6	24.84	0.00
28-Jul-19		X	31.7	26.23	0.00
29-Jul-19	X	X	31.7	26.48	0.00
30-Jul-19		X	29.4	24.34	1.27
31-Jul-19		X	30.6	24.86	0.00
1-Aug-19		X	28.9	24.20	0.00
2-Aug-19		X	30.6	24.86	0.00
3-Aug-19	X		30.6	23.96	0.00
4-Aug-19		X	31.7	23.97	0.00
5-Aug-19		X	31.7	25.67	18.03
6-Aug-19		X	30.0	24.02	0.25
7-Aug-19	X		28.9	24.39	2.79
8-Aug-19		X	31.7	25.26	0.00
9-Aug-19	X		28.3	23.10	0.25
10-Aug-19		X	28.3	21.74	0.00
11-Aug-19			28.9	22.57	0.00
12-Aug-19		X	29.4	24.74	0.00
13-Aug-19			28.3	24.24	7.37
14-Aug-19		X	29.4	23.66	0.00
15-Aug-19			29.4	24.47	0.00
16-Aug-19		X	28.9	24.34	0.00
mean			29.44	23.90	1.27
SD			1.71	1.56	3.83
