# Supplementary material for: Short-term persistence of foliar insecticides and fungicides in pumpkin plants and their pollinators
Source: PLoS One. 2025 Apr 2;20(4):e0311634. doi: 10.1371/journal.pone.0311634 (PMC11964230; doi:10.1371/journal.pone.0311634)
Supplement: S3 Table — Farms and spray dates are as listed in S1 Table. Pesticide residue names are given as their commercial formulations and % active ingredient(s). The rate of application is given in fluid ounces per hectare, either as a range or a single concentration, as reported by the growers. (PDF) [file pone.0311634.s003.pdf]

S3 Table. Additional fungicides applied during the study period that we were not able to test for.

Grower	Spray Event	OxiDate (27% Hydrogen dioxide)	Dithane (7.4% Manganese, 0.9% Zinc, 28.7% ethylene bisdithiocarbamate)	Manzate (15% Manganese, 1.9% Zinc, 58.1% ethylene bisdithiocarbamate)
A	29 Jul	-	-	-
B	29 Jul	-	-	-
	7 Aug	79.1	-	-
C	24 Jul	-	-	-
	9 Aug	-	57.5 – 74.4	-
D	26 Jul	-	-	94.8
	3 Aug	-	-	94.8
E	26 Jul	-	-	-
Farms		1	1	1
Spray Events		1	1	2
