# Supplementary material for: Short-term persistence of foliar insecticides and fungicides in pumpkin plants and their pollinators
Source: PLoS One. 2025 Apr 2;20(4):e0311634. doi: 10.1371/journal.pone.0311634 (PMC11964230; doi:10.1371/journal.pone.0311634)
Supplement: S4 Table — Three risk quotients were calculated for each tissue: HQ (Hazard Quotient), HQPPB (Hazard Quotient in which LD50 was first converted to bee-relevant PPB), and RQ (Risk Quotient from the US EPA BeeREX tool). See Methods for details of index calculations. Bold font indicates the index value exceeded the threshold level of concern (HQ > 50, HQPPB > 1, RQ > 0.40), and, therefore, low risk cannot be concluded. Only pesticides for which data were available were included in this table. For risk assessment based on the maximum chemical concentrations found in each tissue, see main text Table 3. (PDF) [file pone.0311634.s004.pdf]

S4 Table. Risk assessment for bees visiting pumpkin within one week after foliar spray, based on the minimum chemical concentrations detected in each tissue type.

Tissue	Chemical	***HQ***	***HQ_{PPB}***	***RQ***
Leaf	Carbaryl	**88.64**	0.01	**96.06**
	Permethrin	**86319.58**	**10.36**	**67.19**
	Cyhalothrin	**17831.84**	**2.14**	**14.14**
	Triflumizole	15.27	<0.01	0.05
	Chlorothalonil	ND	ND	0.07
Pollen	Carbaryl	**127.29**	0.02	<0.01
	Permethrin	**445.46**	0.05	<0.01
	Cyhalothrin	ND	ND	0.00
	Triflumizole	1.67	<0.01	<0.01
	Quinoxyfen	**267800.00**	**32.14**	0.01
	Chlorothalonil	ND	ND	0.00
Nectar	Carbaryl	**52.43**	0.01	0.02
	Permethrin	ND	ND	<0.01
	Cyhalothrin	ND	ND	0.00
	Triflumizole	ND	ND	<0.01
	Quinoxyfen	ND	ND	<0.01
	Chlorothalonil	ND	ND	0.00
Bee	Carbaryl	**130.06**	0.02	NA
	Permethrin	ND	ND	NA
	Quinoxyfen	ND	ND	NA
	Chlorothalonil	ND	ND	NA
