# Supplementary material for: Short-term persistence of foliar insecticides and fungicides in pumpkin plants and their pollinators
Source: PLoS One. 2025 Apr 2;20(4):e0311634. doi: 10.1371/journal.pone.0311634 (PMC11964230; doi:10.1371/journal.pone.0311634)
Supplement: S5 Table — The percent of samples (rounded to the near integer) in which any one or two different chemicals were detected was calculated separately for insecticides and fungicides using only data from farms where multiple sprays from each category were used. Four out of five farms used a mix of both insecticides and fungicides during the study period, but no farms sprayed all three of our focal insecticides or all three fungicides during the study. Time since foliar application is indicated as -1 = one day before, 1 = one day after, 3 = three days after, 7 = seven days after. (PDF) [file pone.0311634.s005.pdf]

S5 Table. Detection of one versus multiple insecticide and fungicide residues in pumpkin and bee tissues at farms that applied multiple chemicals.

Tissue	Time	One Insecticide	Two Insecticides	One Fungicide	Two Fungicides
Leaf	-1	50	0	0	0
	1	75	50	17	0
	3	50	50	17	0
	7	50	0	17	0
	total	56	25	13	0
Pollen	-1	0	0	14	0
	1	29	0	20	25
	3	14	0	17	25
	7	13	0	17	0
	total	16	0	16	13
Nectar	-1	0	0	0	0
	1	43	0	0	0
	3	17	0	0	0
	7	0	0	0	0
	total	14	0	0	0
Bee	-1	0	0	0	0
	1	8	0	0	0
	total	3	0	0	0
