# Supplementary material for: Short-term persistence of foliar insecticides and fungicides in pumpkin plants and their pollinators
Source: PLoS One. 2025 Apr 2;20(4):e0311634. doi: 10.1371/journal.pone.0311634 (PMC11964230; doi:10.1371/journal.pone.0311634)
Supplement: S8 Table — Time since spray is indicated as -1 = one day before, 1 = one day after, 3 = three days after, 7 = seven days after. The number of samples tested (N) and the number in which triflumizole was detected (NPos.) is given for each time. Minimum and maximum concentrations are in units of μg/ kg (PPB). Risk quotients HQ, HQPPB, and RQpollen and RQnectar are based on the maximum residue concentration. RQleaf is not available by day because it was estimated from initial application rate, not leaf concentration. Bold font indicates concentration exceeded a threshold level of concern of HQ > 50, HQPPB > 1, or RQ > 0.40. (PDF) [file pone.0311634.s008.pdf]

S8 Table. Detection of triflumizole fungicide in pumpkin and bee tissues before a spray event through one week after.

Tissue	Time	N	$N_{Pos.}$	Min	Max	*HQ*	HQ_{PPB}	*RQ*
Leaf	-1	2	0	-	-	0.0	0.0	NA
	1	2	1	9023.1	9023.1	**451.2**	0.1	NA
	3	2	1	5937.8	5937.8	**296.9**	0.0	NA
	7	2	1	305.4	305.4	15.3	0.0	NA
Pollen	-1	2	0	-	-	0.0	0.0	0.0
	1	2	1	23.3	23.3	1.7	0.0	<0.01
	3	2	1	24.7	24.7	1.8	0.0	<0.01
	7	2	0	-	-	0.0	0.0	0.0
Nectar	-1	2	0	-	-	0.0	0.0	0.0
	1	2	0	-	-	0.0	0.0	0.0
	3	1	0	-	-	0.0	0.0	0.0
	7	1	0	-	-	0.0	0.0	0.0
Bee	-1	0	-	-	-	-	-	NA
	1	0	-	-	-	-	-	NA
